# Supplementary figures and images for: Constitutively active STING causes neuroinflammation and degeneration of dopaminergic neurons in mice
Source: eLife. 2022 Oct 31;11:e81943. doi: 10.7554/eLife.81943 (PMC9767458; doi:10.7554/eLife.81943)

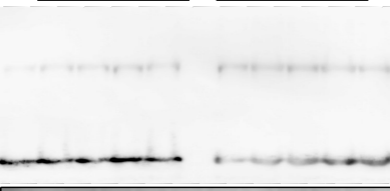

Supplement: Figure 3—figure supplement 1—source data 1. [file elife-81943-fig3-figsupp1-data1.zip › F3S1 source data/F3S1A.pdf]

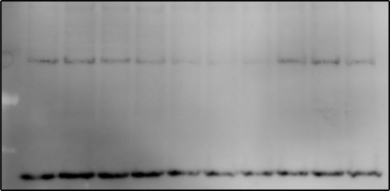

Supplement: Figure 3—figure supplement 1—source data 1. [file elife-81943-fig3-figsupp1-data1.zip › F3S1 source data/F3S1B.pdf]

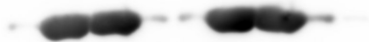

Supplement: Figure 6—figure supplement 1—source data 1. [file elife-81943-fig6-figsupp1-data1.zip › F6S1 source data/F6S1E.pdf]

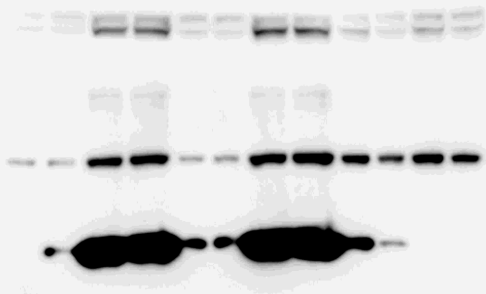

Supplement: Figure 6—figure supplement 1—source data 1. [file elife-81943-fig6-figsupp1-data1.zip › F6S1 source data/F6S1F.pdf]

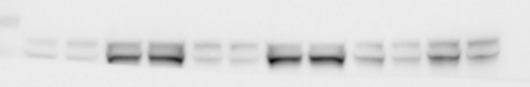

Supplement: Figure 6—figure supplement 1—source data 1. [file elife-81943-fig6-figsupp1-data1.zip › F6S1 source data/F6S1G.pdf]

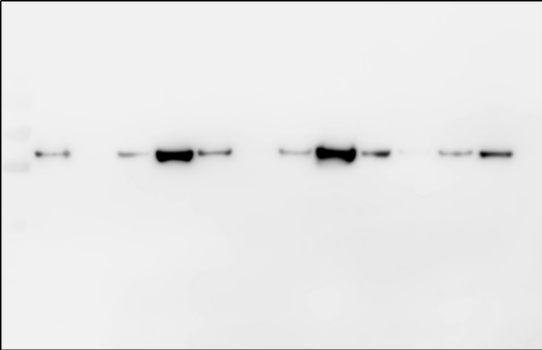

Supplement: Figure 6—figure supplement 1—source data 1. [file elife-81943-fig6-figsupp1-data1.zip › F6S1 source data/F6S1C.pdf]

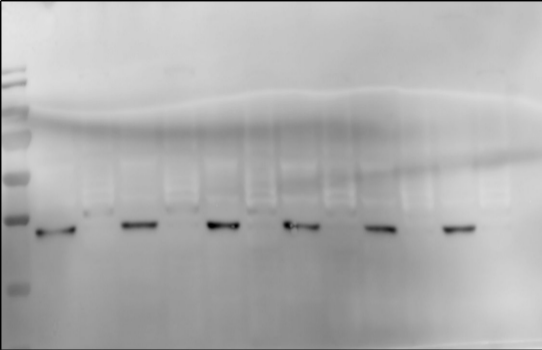

Supplement: Figure 6—figure supplement 1—source data 1. [file elife-81943-fig6-figsupp1-data1.zip › F6S1 source data/F6S1B.pdf]

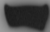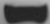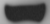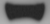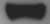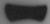

Supplement: Figure 6—figure supplement 1—source data 1. [file elife-81943-fig6-figsupp1-data1.zip › F6S1 source data/F6S1A.pdf]

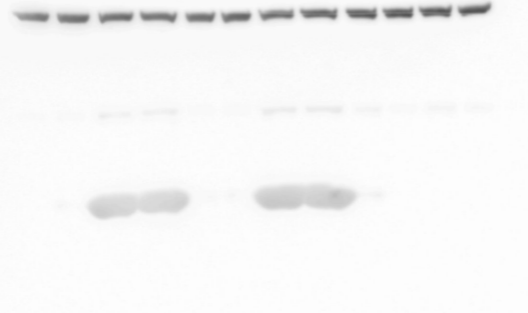

Supplement: Figure 6—figure supplement 1—source data 1. [file elife-81943-fig6-figsupp1-data1.zip › F6S1 source data/F6S1H.pdf]
